# Mutual information and variants for protein domain-domain contact prediction

**DOI:** 10.1186/1756-0500-5-472

**Published:** 2012-08-31

**Authors:** Mireille Gomes, Rebecca Hamer, Gesine Reinert, Charlotte M Deane

**Affiliations:** 1 Department of Statistics, University of Oxford, Oxford, UK

## Abstract

**Background:**

Predicting protein contacts solely based on sequence information remains a challenging problem, despite the huge amount of sequence data at our disposal. Mutual Information (MI), an information theory measure, has been extensively employed and modified to identify residues within a protein (intra-protein) that are in contact. More recently MI and its variants have also been used in the prediction of contacts between proteins (inter-protein).

**Methods:**

Here we assess the predictive power of MI and variants for domain-domain contact prediction. We test original MI and these variants, which are called MIp, MIc and ZNMI, on 40 domain-domain test cases containing 10,753 sequences. We also propose and evaluate two new versions of MI that consider triangles of residues and the physiochemical properties of the amino acids, respectively.

**Results:**

We found that all versions of MI are skewed towards predicting surface residues. Since domain-domain contacts are on the surface of each domain, we considered only surface residues when attempting to predict contacts. Our analysis shows that MIc is the best current MI domain-domain contact predictor. At 20% recall MIc achieved a precision of 44.9% when only surface residues were considered. Our triangle and reduced alphabet variants of MI highlight the delicate trade-off between signal and noise in the use of MI for domain-domain contact prediction. We also examine a specific “successful” case study and demonstrate that here, when considering surface residues, even the most accurate domain-domain contact predictor, MIc, performs no better than random.

**Conclusions:**

All tested variants of MI are skewed towards predicting surface residues. When considering surface residues only, we find MIc to be the best current MI domain-domain contact predictor. Its performance, however, is not as good as a non-MI based contact predictor, i-Patch. Additionally, the intra-protein contact prediction capabilities of MIc outperform its domain-domain contact prediction abilities.

## Background

Proteins are actors in a complex system, with their functions to a large extent defined by their interactions with other proteins. It is the size, shape and chemical properties of the residues on the surface of the protein that dictate the capacity of a protein to interact with other proteins. The ability to predict the residues involved in these interactions would help to identify specific functionality, structural constraints and even disease-causing mutations.

In this paper we examine the capacity for mutual information (MI) methods to predict these contact residues between proteins by assessing their ability to predict contacts between two domains of a protein. To date, MI based methods have been extensively used to predict contacts within a protein (intra-protein) [1-12].

MI uses a multiple sequence alignment (MSA) of homologous sequences; it measures the dependence between two columns in this alignment, with the aim of identifying correlated mutations [13]. If two residues are in close proximity, it is likely that a change in size, shape or chemistry of one will need to be compensated for by a change in the other, if the contact is to remain energetically favourable [3,14-16]. These compensatory mutations are often referred to as correlated mutations.

MI was first applied to sequence alignments by Korber *et al.* to identify covarying positions in a viral peptide [1]. We hypothesise that MI has since gained popularity because it is non-parametrized, *i.e.* the scores of MI are solely dependent on an MSA and no additional information, such as a phylogeny propensity table [12], a similarity matrix [17,18] and so on is required. Furthermore, unlike other algorithms that predict contact “patches” [7,19], or individual contact residues [20,21], MI attempts to predict specific pairs of residues that are in contact with one another.

Horner *et al.*[22] have collated the accuracies of intra-protein contact residue prediction from several publications that employ correlated mutation analysis algorithms, and showed that MI has an accuracy between 2% and 18%. Accuracy here refers to the percentage of predictions that are correct. The low accuracy of MI has in turn precipitated many variants that attempt to improve its performance. These variants specifically attempt to correct for the following three recognised limitations of MI: highly variable columns, phylogenetic relationships and insufficient sequences in the MSA. There is evidence that columns in the MSA that have a high variability contribute to random and non-random high MI scores [8,23], while phylogenetic relationships [5] and insufficient number of sequences in the MSA [8] weaken the signal detection ability of MI. In 1995, Clarke [2] corrected the MI score by a measure relating to the number of amino acid pairs occurring at each position to negate the influence of highly diverged sequences that may be inappropriately aligned in the MSA. Later, Wollenberg and Atchley [5] used parametric bootstrapping to adjust for evolutionary relationships; another technique for reducing phylogenetic noise in the MSA before employing MI is the evolutionary trace approach [24]. Tillier and Lui [6] designed a tool which removes columns in an MSA that carry a high phylogenetic signal and then employs MI to identify positions in the resulting MSA that coevolve with each other, but do not coevolve significantly with other positions. As performance was still disappointing, Martin *et al.* attempted to remove the noise caused by entropy by dividing the MI score of a pair of columns by their joint entropy [8]. These authors also suggested that a minimum of 125 sequences should be used in an MSA to reduce stochastic noise. Dunn *et al.* improved on this score by introducing MIp, which modified the MI value by a measure that aims to eliminate phylogenetic and entropic effects [9]. Subsequently, Lee and Kim [25] introduced two other powerful phylogenetic noise reduction MI measures, MIc and aMIc. In 2010 Brown and Brown [11] suggested yet another MI measure, ZNMI, that accounts for different alphabet sizes among columns in the MSA. These authors also proposed a pipeline to yield highly reproducible scores. Despite all of these efforts, to date no single MI measure has achieved general utility or wide acceptance for predicting intra-protein contact sites.

MI has begun to be extended to predict inter-protein contact residues. Halperin *et al.*[26] carried out a small study of original MI and other correlation algorithms on 15 bacteria and archea fusion protein families, and Lee and Kim [25] evaluated their newly formulated MI measures on a specialised dataset of 27 homo-trimers. There have also been several high profile case studies on a small number of examples (one to three), such as [8-11,27]. However, so far there has been no systematic study on a large, general purpose, cross-species dataset of the performance of MI and its latest variants on inter-protein contact residue prediction, partly because no such large inter-protein dataset with accurately paired MSAs is available [25,26].

Following the idea of Pazos *et al.*[18], here we make a step towards closing this gap by employing MI and its variants on 40 domain-domain test cases; the contacts between domains serve as a proxy for inter-protein contacts [18]. We evaluate MI measures that do not require any additional information and rely solely on the sequence alignment itself; we focus on the original MI and its most recent extensions MIp and MIc, as well as ZNMI. Unlike the other measures, the ZNMI score is embedded in an iterative pipeline designed by its authors [11]. Hence we have to analyse the performance of ZNMI differently to its competitors.

We have also attempted to strengthen the predictive capabilities of MI by introducing two new MI variants. The first variant considers triangles of residues rather than pairs to identify contacts, with the aim of enhancing the signal, and is referred to as MI3D and MIp3D. As MIc already considers a third column in its normalising term, it was not extended to triangle scores. Our second variant is designed to reduce noise by grouping residues in the MSA into seven physiochemical categories and subsequently calculating MI. This modification is indicated by the suffix RA (reduced alphabet), and the resulting five variants are: MIRA, MI3DRA, MIpRA, MIp3DRA and MIcRA. Thus altogether we examine the domain-domain contact prediction ability of 10 MI measures: MI, MI3D, MIRA, MI3DRA, MIp, MIp3D, MIpRA, MIp3DRA, MIc and MIcRA, alongside the pipeline ZNMI.

In our study we observe that all versions of MI do not perform as well as a current leading domain-domain contact predictor, i-Patch [12], which achieves a precision of 48.9% at 20% recall on the 40 test cases. The enhanced predictive ability of i-Patch may arise from its use of surface residues only, residue propensity scores, as well as the MSA. Conversely, MI variants rely solely on the MSA. Upon further investigation we find that MI, MIp and MIc scores are skewed towards predicting surface residues rather than contact. Thereafter, like i-Patch, we consider surface residues only when attempting to predict domain-domain contacts using MI. This in turn improves the contact prediction abilities of all 10 MI measures. Amongst the 10 tested MI variants and ZNMI, we find that MIc is the leading MI domain-domain contact predictor, attaining a precision of 44.9% precision at 20% recall.

## Results and discussion

For this inter-protein MI investigation we use 40 proteins that have two domains, and treat each domain as a separate protein. Proteins are composed of one or more domains. A multidomain protein, protein A, will often use one of its domains to bind to protein B and another to bind to protein C, thus allowing protein A to perform multiple functions. Consequently, in reality protein-protein interactions are often domain-domain interactions [28]. Hence using domain-domain interactions within proteins as a proxy for protein-protein interactions ensures that interacting “proteins” are accurately paired in each MSA, while capturing interaction mechanisms.

Unfortunately the seemingly more straightforward approach of building MSAs by finding homologous sequences of two proteins known to be in complex, and then pairing the sequences originating from the same species, is not feasible for the following reasons. Inferring protein-protein interactions across species based on sequence homology has a low level of accuracy, requiring a sequence identity of far higher than 70% [29]. Using only sequences with >70% identity would have resulted in MSAs with a low number of sequences and few amino acid changes, not sufficient enough to yield statistically significant MI results [8]. Furthermore, within a species there may be multiple homologs of the interacting proteins, and selecting the correct pairs out of this is an unsolved problem [29]. Therefore previous protein-protein contact residue prediction investigations have chosen to employ proteins of known structure, with two domains, that have several homologous sequences available [12,18]. Hamer *et al.*[12] have shown that the propensities of amino acids to occur as contact residues between two domains in a protein and between protein complexes are highly similar. While it is hence plausible to use domain-domain interactions as a proxy for inter-protein interactions, it is however possible that protein-protein interfaces may indeed differ from domain-domain interfaces.

Here we want to predict whether a residue is a contact residue or not using MI-based scores on the generated domain-domain MSAs. As MI assigns scores to pairs of columns in an MSA, first we calculate the MI score for all pairs of columns, or triangles of columns in the case of the 3D variants. To obtain a score for individual columns, each residue in all 40 test cases was assigned the maximum MI score that the residue column achieved with any other residue column in its MSA. We also tested assigning the average score of each residue column, but this resulted in a significant decrease in the performance of the MI variants. A residue is then assigned the score of its column.

When calculating MI pair or triplet scores, as in previous work [9,25], only ungapped aligned columns were used. When allowing gapped columns there was a tendency for MI methods to underperform.

For the 40 test cases employed, the probability of randomly selecting contacts, *i.e.* correctly picking a contact residue from the total set of residues, without any information about the proteins involved, is 17.1%. Running i-Patch [12], a non-MI-based domain-domain contact predictor, on this dataset resulted in a precision of 48.9% at 20% recall. When assessing domain-domain contact prediction, Lee and Kim [25] found that their MIc measure outperformed their additionally normalised aMIc score [25], as well as Dunn *et al.*’s MIp measure [9]. We also found MIc to be the best domain-domain contact predictor of our tested MI variants. On our 40 test cases, MIc attained a precision of 34.7% at 20% recall, demonstrating that the performance of MI methods is below that of the parametrised method i-Patch.

We conjecture that the enhanced classification capabilities of i-Patch may be due to its use of residue propensities, along with its consideration of only residues on the surface of a protein when attempting to predict contacts between proteins. Consequently, we examined the effect of surface *versus* buried residues on domain-domain MI contact prediction.

In our 40 test cases, the probability of randomly selecting a surface residue from all residues is 69.9%. Using MI, MIp and MIc on our dataset as surface residue predictors (is the highest scoring residue on the surface?), we observed that each of the measures surpassed this random classification and achieved a precision of 86.9%, 75.5% and 74.1% respectively, at 20% recall (Additional file 1: Figure S1). Thus it appears that high scores of all three variants of MI are skewed towards surface residues. This is probably due to the observed high entropy of surface residue columns.

**Figure 1:**
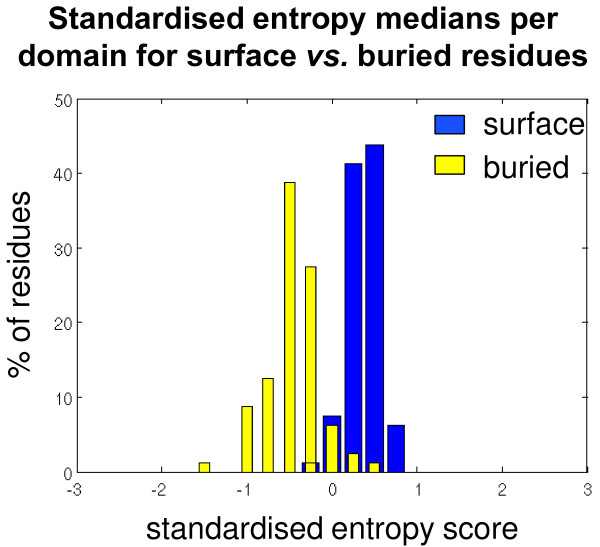
**Standardised entropy medians of surface *versus *buried residue columns for all domains in the dataset.** Comparing the medians of the standardised entropy scores of each domain’s surface residue columns (blue) against the medians of each domain’s buried residue columns (yellow). Residue columns containing one or more gaps, or having an entropy score of 0 are not included in the median calculation.

Prior investigations have shown that MI scores strongly correlate with the entropy of the columns involved [8,23]. Figure 1 shows that MSA columns corresponding to surface residues tend to have a higher entropy than those associated with buried residues. The observed lower column entropy for buried residues is consistent with previous studies that have shown that buried residues are under greater evolutionary constraints than solvent-accessible surface residues [30-33]. A slower rate of evolution of these residues is unsurprising since buried residues often play a crucial role in maintaining the 3D structure of a protein. We hypothesise that this skewness of MI towards surface residues in turn perturbs its ability to predict contact residues. With this in mind we eliminated buried residues from further evaluation of the performance of MI, MIp and MIc for domain-domain contact prediction.

**Figure 2:**
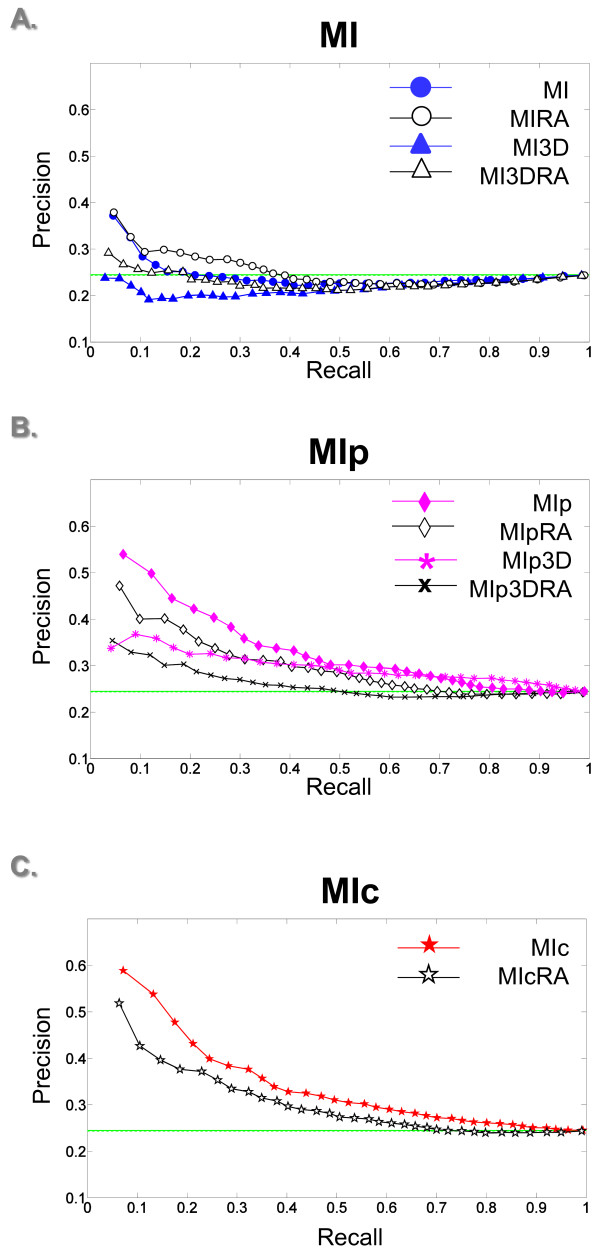
**Contact *versus *non-contact prediction P-ROC curves for MI variants on the 40 test cases. A**, **B** and **C** illustrate the performance of MI, MIp and MIc variants respectively when distinguishing contact from non-contact surface residues. The solid green line in all plots depicts the chance of randomly selecting a contact residue, while the dashed green line indicates the probability of randomly selecting a contact residue when employing the reduced alphabet amino acid set.

After filtering out buried residues in the 40 test cases, the precision of MIc increases from 34.7% to 44.9% at 20% recall (Figure 2C). The probability of randomly selecting a contact residue is now 24.4%. Excluding buried residues therefore clearly has a considerable effect. As can be observed in Figure 2, Table 1 and Additional file 2: Figure S2, MIc still outperforms the other MI variants. MI and MIp achieve a precision of 24.4% and 42.3% respectively at 20% recall (Figures 2A and B, and Table 1).

**Table 1:** Precision for detecting contact *versus* non-contact residues at 20% recall

**Contact prediction**	
**MI variant**	**Precision contact *vs.* non-contact**
MIc	44.9
MIp	42.3
MIcRA	36.9
MIpRA	35.8
MIp3D	31.8
MIp3DRA	29.4
MIRA	28.4
Random	24.5
MI	24.4
RandomRA	24.4
MI3DRA	23.5
MI3D	19.9

Results are given for the 40 test cases. MI variants are listed in descending order of contact *versus* non-contact precision, *i.e.* best to worst classifier of contact residues. The probability of randomly selecting a contact residue from all surface residues is 24.5%. This probability changes to 24.4% when using the reduced alphabet amino acid set because residues are lost as the entropy of their corresponding MSA column reduces to 0.

For a meaningful comparison of scores however, we need to assess their stability. In order to test this we randomly selected 70% of the sequences from each MSA 100 times, and recalculated the variant MI scores for each of the sub-alignments (Table 2). We observed that the rank order of the top five MI variant scores was maintained (Table 2). This sub-alignment creation and MI recalculation procedure was only carried out on those 24 test cases that had ≥200 sequences, to ensure that there would be at least 125 sequences in the sub-alignments, the suggested minimum number of sequences required to reduce the stochastic noise in the MSA [8]. Hence the results in Table 1 refer to 40 test cases, while those in Table 2 reflect the mentioned subset of 24 cases. Based on two-sample t-tests, with a sample size of 24, the differences between the top four scores in Table 2 are highly significant at the 0.1% level.

**Table 2:** Precision for detecting contact *versus* non-contact residues at 20% recall, for sub-alignments of 70%

**Contact prediction for sub-alignments**			
**MI variant**	**70% AVG**	**70% STD DEV**	**100%**
MIc	52.5*	2.1	54.8
MIp	46.0*	2.1	47.4
MIcRA	41.9*	1.8	41.4
MIpRA	38.2*	1.5	38.5
MIp3D	30.6	1.3	28.5
MIRA	30.2*	1.2	31.4
MIp3DRA	28.0*	1.2	30.9
MI	25.8*	0.8	27.6
MI3DRA	23.2*	1.0	25.5
Random	-	-	24.4
RandomRA	-	-	24.4
MI3D	20.0	0.6	21.8

70% of sequences in an MSA were randomly selected and the 10 MI variant scores based on the new sub-alignment were calculated. This subset selection and calculation procedure was repeated 100 times for those test cases that had ≥200 sequences to ensure ≥125 sequences in each sub-alignment [8]. Thus 24 test cases were used. For each of the 100 iterations a P-ROC curve similar to Figure 2 was plotted for the 24 test cases (figures not shown), and the precision at 20% recall recorded. Columns one and two, respectively, contain the averages and standard deviations of these 100 precision values. Column three indicates the precision attained at 20% recall when all sequences in the 24 original MSAs were used. When considering this set of 24 cases, the probability of randomly selecting a contact residue from all surface residues is 24.4% generally and when using the reduced alphabet MSAs. The MI variants are listed in descending order of the average precision of the 100 70% sub- alignments. The presence of an ‘*’ at an MI variant indicates that the difference between the precision of this MI variant and the next lowest is significant at the 0.1% level, when using two-sample t-tests with a sample size of 24.

Additionally, it is worth noting that the performance of all non-3D MI variants improve when using MSAs that have ≥200 sequences (Tables 1 and 2).

To account for the mentioned variability in scores due to changes in the MSA, Brown and Brown [11] have designed a new MI measure, ZNMI, as well as a methodology to yield highly reproducible and accurate contact pair prediction scores. Their suggested algorithm repeatedly partitions the MSAs into 50% sub-alignments, calculates the pair scores, retains significant scoring pairs for each partition and subsequently compares all partitions to acquire consensus pair scores. It should be noted that unlike our methodology, this pipeline does not filter out buried residues. The authors provided us with code for MI [8], MIp [9], OMES [23,34], SCA [35], ZNMI [11] and ZRES [10] measures wrapped within their proposed pipeline, but unfortunately not for MIc. Having run this code on our 40 domain-domain test cases we find that using ZNMI in conjunction with their algorithm does improve on the performance of original MI; at 20% recall the precision of ZNMI is 30.5% (Table 3), as opposed to the 24.4% precision of original MI (Figure 2A and Table 1). ZNMI within the Brown and Brown pipeline even outperforms MIp, when MIp is incorporated into the same pipeline (27.1% precision at 20% recall; Table 3). However, the performance of MIp independent of the pipeline, after filtering out buried residues and columns with one or more gaps, supersedes ZNMI and all other coevolving residue algorithms tested by the authors, as illustrated by its precision of 42.3% at 20% recall (Table 3).

**Table 3:** Precision at 20% recall of contact prediction algorithms used within Brown and Brown [11] pipeline

**Brown and Brown**[11]**pipeline**	
**Algorithms**	**Precision contact *vs.* non-contact**
MIp - original, minus buried	42.3
SCA	31
ZNMI	30.5
ZRES	28.9
MIp	27.1
MI	25.7
OMES	25.7
MI - original, minus buried	24.4

Results are given for the 40 test cases. The Brown and Brown [11] pipeline was applied to the contact residue prediction algorithms listed in column one, with the exceptions of MIp and MI “original, minus buried.” As in Table 1, these two algorithms were run independently of the pipeline and buried residue columns, residue columns with one ore more gaps or an entropy of 0 were filtered out. The table is arranged in descending order of precision.

### 3D and Reduced Alphabet MI adjustments

To investigate methods that might further enhance the predictive power of MI variants we designed two adjustments. The first adjustment considers triangles of columns rather than pairs, based on the idea that interactions occur in patches [36]. This variant is denoted by the suffix 3D. The second adjustment, suffixed RA, reduces the 20 amino acids to seven categories based on their physical and chemical properties, with the aim of reducing noise.

The idea behind the 3D version is that protein binding involves patches of residues in contact. This idea has been previously used to predict contact residues [12,36]. Furthermore, the success of MIc lies in its normalising factor, the coevolutionary pattern similarity (CPS) score, which estimates the coevolutionary relationship between the pair of residues currently under consideration and all other residues in the MSA [25]. We thus speculated that adding additional residue information to MI and MIp pair scores may enhance their domain-domain contact predictive capabilities. Hence we created new versions of MI and MIp that consider triangles rather than pairs of columns to identify contacts (MI3D (Equation 13) and MIp3D (Equation 14)). Increasing the dimensionality of MI and MIp in this manner surprisingly worsened performance in both cases; the precision at 20% recall of MI3D and MIp3D are 19.9% and 31.8% respectively as compared to precision of MI and MIp of 24.4% and 42.3% (Figure 2 A and B, and Table 1). We conjecture that adding an extra dimension to MI and MIp magnifies the noise in the MSA more than it boosts the signal.

Assuming that contact residues mutate in a correlated manner in order to maintain their interaction, it is not evident how much of a change a residue can undergo while still maintaining its contacts. Using a reduced alphabet residue set addresses this point; as it groups residues by their physiochemical properties, under the assumption that residues with the same physiochemical properties will maintain similar interactions. Grouping the 20 amino acids into seven categories only improved the performance of basic MI and MI3D, which rose in precision at 20% recall from 24.4% to 28.4% and 19.9% to 23.5% respectively (Figure 2A and Table 1). In all other cases the reduced alphabet (RA) appeared to reduce noise as well as signal (Figure 2, Table 1 and Additional file 2: Figure S2).

### Case study

The case study Skerker *et al.*[27] has received a lot of attention for successfully determining inter-protein contact specificity residues with the aid of MI. The authors used original MI (Equation (4)) to determine a subset of contact residues that allow for specific binding of a histidine kinase (HK) with its interacting response regulator (RR). The MSA provided by these authors does not contain the sequence of the structure used in their analysis. Hence we ran MI and MIc on the HK-RR MSA provided by Hamer *et al.*[12], which does include the sequence of this reference structure.

As Skerker *et al.* were interested in residue pairs only between the DHp domain (four helix bundle) of the HK and its interacting RR, only these MI and MIc scores were considered when examining performance. In accordance with our evaluation method on the 40 test cases, all buried residues were eliminated, as were residues corresponding to columns that had one or more gaps or an entropy of 0. This leaves us with 46 DHp residues, nine of which are contacts, and 68 RR residues, amongst which 24 are contacts.

As we have no score cut-off for predictions we check the number of correct predictions among the top nine predictions for DHp. If there was no relationship between the MI scores and contact sites, then the number of correct predictions would follow a Binomial distribution with sample size nine and probability of success 9/46. Under this model we would expect 1.76 correct predictions.

For RR there are 24 contact residues. We check the number of correct predictions among the top 24 predictions. If there was no relationship between the MI scores and contact sites, then the number of correct predictions would follow a Binomial distribution with sample size 24 and probability of success 24/68. Under this model we would expect 8.47 correct predictions.

The results are recorded in Table 4. The p-value is the probability of seeing a number this large or larger under the corresponding Binomial model. None of the p-values are below 5%. Therefore at the 5% level there is no statistical evidence to reject the null hypothesis that in this case study random guess does as well as MI and MIc.

**Table 4:** Performance of MI and MIc on a histidine kinase (HK) - response regulator (RR) complex

**Case study**				
	**DHp: 9 contacts out of 46**		**RR: 24 contacts out of 68**	
	**Contacts among top 9**	**p-value**	**Contacts among top 24**	**p-value**
MI	3	0.2503	9	0.4864929
MIc	4	0.0800	9	0.4864929

MI and MIc are run on the HK-RR MSA provided by Hamer *et al.*[12]. Each surface, ungapped DHp residue column, having a column entropy greater than 0, is assigned the maximum score it achieves when paired with the RR residue columns. These DHp residues are then ranked according to score and the number of true contact residues amongst the top nine scores are recorded for MI and MIc respectively. The same steps are applied to residues in the RR and the number of true contact residues in the top 24 scores are counted for MI and MIc. The p-value refers to the probability of seeing a number this large or larger under the corresponding Binomial model.

## Conclusions

MIc is the best current MI domain-domain contact predictor. The performance of MIc on our domain-domain test cases is not as good as its intra-protein contact prediction [25]. Its predictive capabilities are also not as high as i-Patch [12], a non-MI-based domain-domain contact predictor, but unlike this algorithm, MIc relies solely on sequence information in an MSA. Our 3D and reduced alphabet variants of MI did not improve prediction, but illustrate the delicate trade-off between signal to noise in the use of MI for domain-domain contact prediction.

## Methods

In order to perform the tasks in our methods, code was written in perl, MATLAB and Python, and is available upon request.

### Datasets

For this domain-domain MI investigation we use proteins that have two domains, rather than protein complexes, and treat each domain as a separate protein. In this manner we can be sure that accurate “protein-protein” pairings are used in the MSA. The MSAs are taken from [12], available at http://www.stats.ox.ac.uk/research/bioinfo/resources, which in turn are based on datasets in [18,37-39]. The proteins for which each MSA was constructed has a known pdb structure [40] of X-ray resolution 2.5Å or better, and well annotated domain boundaries [12]. This structure is henceforth referred to as the “reference structure” and is used to identify surface, buried, contact and non-contact residue columns within the MSA. The MSA was generated by using the structural protein as a BLAST query [41,42] against the NCBI-NR database [43]. The homologs identified were made non-redundant at the 90% level using Cd-hit [44]. The final alignment was generated using MUSCLE [45] and MaxAlign [46].

Amongst the set of 67 protein cases available from [12], proteins that contain a single domain that interacts with more than one other domain in the set are disregarded. We chose to omit these proteins as domains interacting with multiple domains may have undergone correlated mutations not pertaining to the pair of domains being presently considered. We thus lose 15 of the 67 cases. In order to aid statistical analysis of the results we select only those domain pairs that have at least 20 contact and 20 non-contact residues on each domain, and the corresponding MSA columns of these residues must be ungapped and have an entropy greater than 0. Therefore a further 9 test cases are lost. Similarly, we filter out 1 test case that has less than 20 surface and buried residues respectively. These factors, along with eliminating 2 cases that have poorly annotated secondary structures in their reference pdb structure file [40], leave us with 40 inter-domain MSAs (Table 5; using [12,47]). The 80 single domains in this dataset range in size from 60 to 376 residues.

**Table 5:** The dataset

**Dataset**				
**Protein**	**D1**	**D2**	**Sequences**	**Species**
1A45	1 82	83 173	160	E(146)N(14)
1BIB	67 270	271 317	236	A(12)B(201)N(23)
1BKS	1 188	189 268	478	A(21)B(401)E(10)N(46)
1FNB	19 152	153 314	58	B(22)E(34)N(2)
1G8A	1 51	52 227	75	A(47)E(20)N(8)
1G8P	18 216	261 350	230	A(10)B(143)E(49)N(28)
1I39	1 158	159 200	688	A(32)B(538)E(7)V(1)U(1)N(109)
1J5X	2 169	170 319	252	A(9)B(183)E(5)N(55)
1LAP	1 147	148 484	454	A(2)B(331)E(84)N(37)
1LLD	7 148	149 319	709	A(33)B(389)E(221)N(66)
1MRI	1 162	163 246	68	B(2)E(65)N(1)
1PII	1 255	256 452	75	B(65)N(10)
1RHD	1 156	157 293	505	A(26)B(365)E(57)U(1)N(56)
1THM	1 127	128 208	106	A(1)B(62)E(34)N(9)
1W98	88 227	228 357	70	E(64)N(6)
1WRU	3 176	177 346	64	B(58)V(2)N(4)
1X2G	1 246	247 337	224	A(2)B(155)E(42)N(25)
2AAA	1 376	377 484	245	B(141)E(74)N(30)
2AHE	16 108	109 253	144	B(25)E(100)N(19)
2D3V	3 95	96 195	77	E(71)N(6)
2D8N	16 97	102 189	240	E(195)N(45)
2E64	1 188	189 235	294	A(9)B(231)E(4)U(1)N(49)
2I00	10 300	301 406	116	A(2)B(80)N(34)
2IU5	1 71	72 180	65	B(56)N(9)
2NPO	3 76	77 188	224	A(3)B(182)U(1)N(38)
2NRC	1 247	261 480	188	A(9)B(96)E(68)N(15)
2OF7	17 67	68 207	204	B(135)N(69)
2OI8	8 86	87 216	215	B(151)N(64)
2PGD	1 172	178 433	317	B(211)E(78)N(28)
2PGE	3 136	137 368	138	A(6)B(102)E(1)N(29)
2PGX	2 56	57 250	102	B(87)N(15)
2PHZ	20 142	143 296	420	A(4)B(343)N(73)
2QY9	201 284	285 495	471	A(32)B(344)E(15)N(80)
2REB	23 268	269 328	482	B(434)E(12)N(36)
2TS1	1 220	248 319	598	B(512)E(34)N(52)
4ENL	1 126	127 436	649	A(32)B(448)E(122)N(47)
4MDH	1 154	155 333	339	A(6)B(173)E(134)N(26)
5FBP	1 201	202 335	355	A(3)B(213)E(112)N(27)
6GST	1 82	90 217	374	B(10)E(312)N(52)
8TLN	1 135	136 316	44	A(1)B(36)E(2)N(5)

The “protein” column contains a list of pdb identifiers [40]. D1 and D2 columns denote the start and end pdb residues of domains 1 and 2, respectively. For all pdbs listed, the start and end residues are located in chain A of the structure, except for pdb 1W98 where the mentioned domains are in chain B, and pdb 8TLN in chain E. The “sequences” column indicates the number of sequences present in the multiple sequence alignment (MSA). The final column states the distribution of sequences in each MSA taken from the various species’ domains: eukaryotes (E); archea (A); bacteria (B); viruses (V); unclassified (U); and not found (N), *i.e.* those sequences that could not be found in the NCBI Taxonomy Database. This dataset was taken from Hamer *et al.*[12].

Within the 40 inter-domain MSAs there are non-standard amino acid entries, such as B, Z, X, * and ?. As there is no established method of processing these sequencing uncertainties, we choose to treat them as a gap, while the Brown and Brown pipeline code processes them as additional amino acids [11].

### Identifying the surface *versus* buried residue pairs

For each reference structure protein in the dataset, we calculate the solvent accessibility of the residues using JOY [48]; each domain is treated as a separate entity. In the reference structure, residues that are >7*%*accessible to a 1.4Å radius water molecule are denoted as “surface” residues [48]. Those that do not meet this criterion are termed “buried.” This information about a residue is then annotated to the entire MSA column to which it belongs.

Employing this criterion on our 40 test cases, along with eliminating residue columns that have an entropy of 0 or contain a gap, leaves us with 5483 surface residues and 2364 buried residues. These numbers decline to 5362 and 2174 respectively when employing the 40 reduced alphabet MSAs, as the reduced alphabet MSAs have a greater number of columns with 0 entropy (Equation (1)). The ratios of surface to buried residues in the reduced and non-reduced alphabet sets are 2.5 and 2.3 respectively. The number of 0 entropy columns in the reduced and non-reduced alphabet set are 668 and 326 respectively, while the number of columns with one or more gaps in both sets are 3479.

### Identifying the contact *versus* non-contact residues

**Figure 3:**
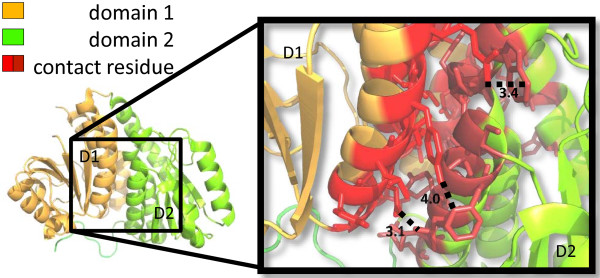
**Contact residues in a pair of interacting domains.** Test case 1J5X.pdb [40]. The two structurally defined domains are depicted in orange (residue 2 to 169) and green (residue 170 to 319) respectively. In the magnified frame, residues in red denote contact residues. Dotted lines and corresponding numbers indicate the Ångström distance between a pair of atoms in the connected residues.

Residues within the binding interface of a pair of interacting domains are labelled as “contact” residues (Figure 3). There is not one particular accepted definition for contact residues [26,49-51]. Here we classified a residue in the representative protein structure as a “contact” residue if: 

1.It is on the surface of the individual domain.

2.It is < 4.5Å from a residue in the other domain [50].

3.The solvent accessibility of the residue is different depending on whether the domain is viewed as a separate structural entity or whether the domain is in complex.

If not all of the above criteria are met, a residue is denoted as a “non-contact” residue.

Using these criteria, and once again ignoring residue columns having an entropy of 0 or a gap, leaves us with 1342 contact and 4141 non-contact surface residues, over all 40 test cases. These numbers decline to 1306 and 4056 respectively when employing the reduced alphabet on the 40 test cases. The ratio of contact to non-contact residues is 0.32 in both the reduced and non-reduced alphabet sets.

### Calculating the Shannon Entropy

The entropy *H*_*unstandardised*_(*J*) of each column *J* in an MSA is calculated by Equation (1) with *log* denoting *log*_*e*_ here,

(1) $$\;H_{\text{unstandardised}}(J)=-\overset{n}{\underset{i=1}{\sum }}P(J=j)\text{log}P(J=j).$$

We use *log*_*e*_ in our MI calculations so that we may compare our results with other MI investigations [23,27]. In this equation *J* is a column in the MSA with probabilities *P*(*J* = *j*) for the discrete set of *n* amino acids *jε*{1,…,*n*}. When *P*(*J* = *j*) = 0 then we set *P*(*J* = *j*)log *P*(*J* = *j*) = 0. The entropy is maximal when all *j* are equally likely to occur, *i.e.P*(*J* = *j*) = 1/*n *and $H_{\text{unstandardised}}(J)=-\sum \frac{1}{n}\text{log}\frac{1}{n}=\text{log}n$[13].

In order to compare the entropies from different MSAs we standardise the entropy score as follows:

(2) $$\;H(J)=\frac{H_{\text{unstandardised}}(J)-{\bar{H}}_{\text{unstandardised}}}{\sigma_{H_{\text{unstandardised}}}},$$

where *H*_*unstandardised*_(*J*) is the entropy of column *J* in the MSA, and ${\bar{H}}_{\text{unstandardised}}$ and $\sigma_{H_{\text{unstandardised}}}$ are the average entropy and estimated standard deviation, respectively, over all columns in the MSA combined. Our calculated entropies range from 0.0 to 2.8, while the standardised entropies vary from -4.2 to 3.0.

### Calculating MI

The joint entropy of two columns *J* and *K* is defined as:

(3) $$\;\begin{array}{c} H_{\text{unstandardised}}(J;K)=\\ \;\;-\overset{n}{\underset{j=1}{\sum }}\overset{m}{\underset{k=1}{\sum }}P(J=j,K=k)\text{log}P(J=j,K=k), \end{array}$$

where column *J* has *n* different residues, and column *K* has *m* different residues.

The general MI formula is:

(4) $$\;\begin{array}{c} \text{MI}{\left(J;K\right)}_{\text{unstandardised}}\;=\;H_{\text{unstandardised}}(J)\\ \;\;\;\;\;+H_{\text{unstandardised}}(K)\\ \;\;\;\;\;-H_{\text{unstandardised}}(J;K). \end{array}$$

The MI is maximal when residues in columns *J* and *K* always covary, *i.e.P*(*J* = *K*) = 1 making the $\text{MI}=-\overset{n}{\underset{j=1}{\sum }}P(J=j)\text{log}P(J=j)$. The maximum MI that can be observed for protein sequences, which have 20 varying residues, is *log*20 ≃ 2.9957 [13].

Unstandardised MI values of 0 are omitted from any further analysis. The reason for this explained in the section titled “MI scores of 0.” The average MI and estimated standard deviation of the MI of all contact and non-contact pairs in the protein are then calculated. A “standardised MI score” is calculated as

(5) $$\;\text{MI}(J;K)=\frac{MI_{\text{unstandardised}}(J;K)-{\bar{\text{MI}}}_{\text{unstandardised}}}{\sigma_{MI_{\text{unstandardised}}}},$$

where *MI*_*unstandardised*_(*J*;*K*) is the MI of columns *J* and *K* in the MSA, and ${\bar{\text{MI}}}_{\text{unstandardised}}$ and $\sigma_{MI_{\text{unstandardised}}}$ are the average MI and estimated standard deviation respectively, over all interacting domains’ column pairs in the MSA, excluding pairs with an MI value of 0, involving a 0 entropy residue column or a gapped residue column.

Our calculated *MI*_*unstandardised*_ scores vary from 0.0 to 1.6, while the standardised MI scores range from -3.2 to 3.7.

### Calculating MIp

Dunn *et al.* proposed a variant of MI that aims to correct for background (random and phylogenetic) noise of each pair of columns under consideration, MIp [9] . This MI correction is denoted by the equation

(6) $$\;\text{MI}p_{\text{unstandardised}}(J;K)=MI_{\text{unstandardised}}(J;K)-\text{APC}(J;K),$$

where *MI*_*unstandardised*_(*J*;*K*) is calculated as denoted in Equation (4). As previously, pairs involving a 0 entropy residue column or a gapped residue column, or having an MI score of 0 are ignored. *APC*(*J*;*K*), the average product correction, is a modification term for columns *J* and *K* in the MSA, evaluated as follows:

(7) $$\;\text{APC}(J;K)=\frac{{\bar{\text{MI}}}_{\text{unstandardised}}(J)\;{\bar{\text{MI}}}_{\text{unstandardised}}(K)}{{\bar{\text{MI}}}_{\text{unstandardised}}},$$

where ${\bar{\text{MI}}}_{\text{unstandardised}}(J)$ is the average mutual information for column *J*, ${\bar{\text{MI}}}_{\text{unstandardised}}(K)$ is the average mutual information for column *K*, and ${\bar{\text{MI}}}_{\text{unstandardised}}$ is the overall average mutual information.

As done previously for MI, MIp scores are also standardised (Equation (8)),

(8) $$\;\text{MIp}(J;K)=\frac{\text{MI}p_{\text{unstandardised}}(J;K)-{\bar{\text{MIp}}}_{\text{unstandardised}}}{\sigma_{\text{MI}p_{\text{unstandardised}}}},$$

where *MIp*_*unstandardised*_(*J*;*K*) is the MIp of columns *J* and *K* in the MSA and ${\bar{\text{MIp}}}_{\text{unstandardised}}$ and $\sigma_{\text{MI}p_{\text{unstandardised}}}$ are the average MIp and estimated standard deviation respectively, over all calculated column pairs in the protein.

Our MIp scores vary from 0.0 to 0.4, while the standardised MIp scores range from -3.1 to 7.0.

### Calculating MIc

Lee and Kim designed normalising measures that aim to reduce phylogenetic noise in MI scores [25]. They begin with the coevolutionary pattern similarity score (CPS) that measures the similarity between the MI score patterns of the two residues being considered. It is denoted as follows,

(9) $$\;\text{CPS}(J;K)\;=\;\frac{1}{n\;-\;2}\;\;\underset{L\neq J;K}{\sum }MI_{\text{unstandardised}}(J;L)MI_{\text{unstandardised}}(K;L).$$

Here *MI*_*unstandardised*_(*J*;*L*) is the MI score of the columns *J* and *L*, which is calculated as described in Equation (4). The number of columns in the MSA are denoted by *n*. Since the CPS is the product of two MI scores, it is then normalised by the square root of the mean of all CPS scores.

(10) $$\;\text{NCPS}(J;K)=\frac{\text{CPS}(J;K)}{\sqrt{\frac{1}{n(n-1)}\underset{J,K}{\sum }\text{CPS}(J;K)}}.$$

To adapt the NCPS for domain-domain prediction we consider only those CPS scores that refer to domain-domain column pairs, *i.e.* one column from each protein, and adjust *n* in Equation (10) accordingly. Once again MI values of 0 are ignored, as are 0 entropy and gapped residue columns. This NCPS score is then subtracted from the corresponding MI pair score to yield Lee and Kim’s noise reduced MI variant, MIc.

(11) $$\;\begin{array}{c} \text{MI}c_{\text{unstandardised}}(J;K)\;=\;MI_{\text{unstandardised}}(J;K)\\ \;\;\;\;\;-\text{NCPS}(J;K). \end{array}$$

MIc scores for each protein are standardised in a manner similar to MI and MIp (Equations 5 and 8), so that MIc values from different proteins can be compared.

(12) $$\;\text{MIc}(J;K)=\frac{\text{MI}c_{\text{unstandardised}}(J;K)-{\bar{\text{MIc}}}_{\text{unstandardised}}}{\sigma_{\text{MI}c_{\text{unstandardised}}}},$$

where *MIc*_*unstandardised*_(*J*;*K*) is the MIc of columns *J* and *K* in the MSA and ${\bar{\text{MIc}}}_{\text{unstandardised}}$ and $\sigma_{\text{MI}c_{\text{unstandardised}}}$ are the average MIc and estimated standard deviation respectively, over all column pairs being considered in the protein.

The MIc scores calculated on our 40 test cases range from -0.02 to 0.1, while the standardised scores range from -2.4 to 7.7.

### 3-dimensional (3D) MI and MIp

MI [27] and MIp [9] were adapted to consider triangles of residues;

(13) $$\;\begin{array}{c} \text{MI}3D_{\text{unstandardised}}(J;K;L)\;=\;\overset{n}{\underset{j=1}{\sum }}\overset{m}{\underset{k=1}{\sum }}\overset{s}{\underset{l=1}{\sum }}P(J=j,K=k,L=l)\\ \;\;\;\;\;\times \text{log}\frac{P(J=j,K=k,L=l)}{P(J=j)P(K=k)P(L=l)}, \end{array}$$

where MSA column *J* from domain 1 has *n* different residues, column *K* from domain 2 has *m* different residues, and column *L* from domain 2 has *s* different residues. Residues in the representative protein structure, corresponding to columns *K* and *L*, should be < 4.5Å from each other in order to be considered as being on the same patch in the domain.

MIp3D is defined as

(14) $$\;\begin{array}{c} \text{MIp}3D_{\text{unstandardised}}(J;K;L)\;=\;\text{MI}3D_{\text{unstandardised}}(J;K;L)\\ \;\;\;\;\;\;-\text{APC}3D(J;K;L). \end{array}$$

In this equation *MI*3*D*_*unstandardised*_(*J*;*K*;*L*) is calculated as denoted in Equation (13) and *APC*3*D*(*J*;*K*;*L*) is calculated as

(15) $$\;\text{APC}3D(J;K;L)=\frac{{\bar{\text{MI}3D}}_{\text{unstandardised}}(J)\;{\bar{\text{MI}3D}}_{\text{unstandardised}}(K)\;{\bar{\text{MI}3D}}_{\text{unstandardised}}(L)}{{\bar{\text{MI}3D}}_{\text{unstandardised}}},$$

where ${\bar{\text{MI}3D}}_{\text{unstandardised}}(J)$ is the average 3D mutual information for column *J*, ${\bar{\text{MI}3D}}_{\text{unstandardised}}(K)$ is the average 3D mutual information for column *K*, ${\bar{\text{MI}3D}}_{\text{unstandardised}}(L)$ is the average 3D mutual information for column *L*, and ${\bar{\text{MI}3D}}_{\text{unstandardised}}$ is the overall average 3D mutual information.

In order to compare the 3D mutual information scores between test cases, MI3D and MIp3D scores were standardised in a manner similar to those described in Equations (5), (8) and (12), respectively. Once again MI3D values of 0 were ignored, as were 0 entropy columns and columns containing one or more gaps.

### Reduced Alphabet (RA) MI scores

We grouped the 20 amino acids into the same seven physiochemical categories successfully employed by Hamer *et al.* in their domain-domain contact predictor, i-Patch [12]. These seven categories include: Small (S,G,A,P), Hydrophobic (V,M,I,L,C), Negatively charged (D,E), Aromatic (F,Y,W), Polar (Q,T,N), Favoured Positively-charged (R,H), and Disfavoured Positively-charged (K). These physiochemical groups are abbreviated to S, H, N, A, P, F and D respectively. Hamer *et al.* introduced Favoured and Disfavoured categories because Lysine (K) was found to be rare in protein/domain interfaces (propensity 0.66), while Arginine (R) and Histidine (H) were far more common (propensities of 1.05 and 1.11, respectively) [12].

We replaced the amino acid alphabets in each MSA by their corresponding category abbreviation and recalculated MI, MIp, MIc, MI3D and MIp3D as described above. The five new MI variant scores are referred to as MIRA, MIpRA, MIcRA, MI3DRA and MIp3DRA.

We choose to employ this particular set of seven physiochemical categories as it was successfully used by i-Patch [12] in domain-domain contact prediction. We do not expect another grouping to dramatically improve the predictive capabilities of MI and its variants further.

### P-ROC curves

For classification, each residue in all 40 test cases was assigned the maximum MI score that its residue column achieved with any other residue column in its MSA. When the average score of each residue was assigned instead, the performance of the MI variants decreased significantly, consequently the maximum score was employed.

When there is a disproportionate number of positive *versus* negative cases, P-ROC (Precision Recall Operating Characteristic) curves [52] provide an alternative to ROC (Receiver Operating Characteristic) curves [53] when attempting to evaluate the performance of a classifier. In our 40 test case dataset, contact residues constitute only 24.5% of all residues, thus the P-ROC will be more informative than the ROC curve. To calculate precision and recall the percentiles of the scores are used as cut-offs, where

(16) $$\;\text{precision}=\frac{\text{TP}}{\text{TP}+\text{FP}},$$

and

(17) $$\;\text{recall}=\frac{\text{TP}}{\text{TP}+\text{FN}}.$$

*TP* in these equations denote the number of true positives, *FP* denotes the number of false positives and *FN* symbolises the number of false negatives.

Each P-ROC plot contains a flat horizontal line (green) at $\frac{\text{TP}}{\text{total}\;\text{scores}}$. This line denotes the probability of randomly discriminating positive *versus* negative cases. For example, in Figure 2 the solid green line is at 0.245 because there are 1342 “contact” scores out of 5483 total surface scores in the non-reduced alphabet test set. In this figure the dashed green line is at 0.244 because there are 1306 “contact” scores out of 5362 total surface scores in the reduced alphabet test set. Similarly, in Additional file 1: Figure S1 the solid green line is at 0.699 as there are 5483 “surface” scores out of 7847 total scores in the non-reduced alphabet test set, while the dashed green line is at 0.712 for there are 5362 “surface” scores out of 7536 total scores in the non-reduced alphabet test set.

### Sub-sampling to test stability of MI scores

To test the stability of the 10 MI variant scores under minor changes in the MSA, for each test case 70% of sequences in the MSA were randomly selected and all 10 MI scores recalculated and 10 respective P-ROC curves were plotted. This sub-sampling and calculation process was repeated 100 times per test case for every MI variant. Then the average and standard error of the precision values for the 100 P-ROC curves were calculated for each MI variant. The precision values at 20% recall for each of the MI variants are listed in Table 2.

This sub-alignment creation and MI recalculation process was only carried out on those 24 test cases that had ≥200 sequences to ensure that a minimum of 125 sequences were retained in each sub-alignment, the suggested minimum number of sequences required to reduce the stochastic noise in the MSA [8].

### MI scores of 0

**Figure 4:**
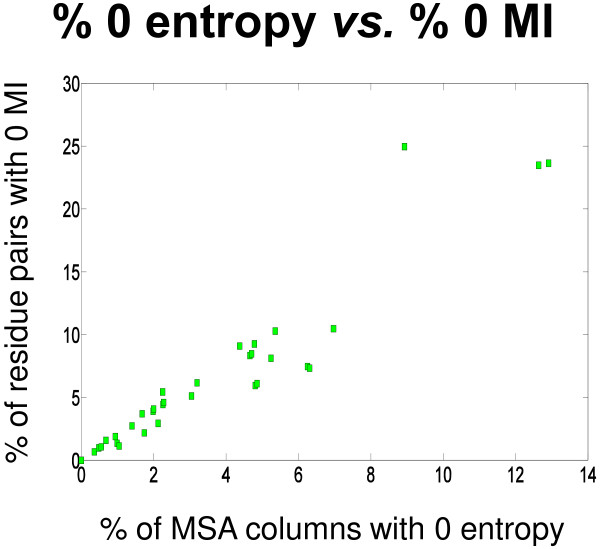
**Effect of entropies of 0 on MI scores.** The percent of columns in an MSA that have an entropy of 0 is plotted against the percent of all domain-domain residue pairs in the corresponding complex that have an MI value of 0. Only those columns in the MSA that correspond to a residue in the reference structure are used. Columns that have one or more gaps are ignored. Each point on the plot represents a single case study in our domain-domain dataset.

Pairing any MSA column with a fully conserved column, *i.e.* a column with an entropy of 0, results in a joint entropy equivalent to the entropy of the non-fully conserved column and subsequently an MI score of 0 for that pair. Since conserved columns do not give any indication of correlated mutations, MI scores involving these columns are ignored. This is standard procedure; for example, [6]. The relationship between percent of columns in an MSA with an entropy of 0 and percent MI scores of 0 computed can be observed in Figure 4. This approximately linear relationship further affirms the direct influence a column with an entropy of 0 has on the MI score of pairs involving that column.

## Competing interests

The authors declare that they have no competing interests.

## Author’s contributions

MG participated in design of the study, wrote the code used for analysis and drafted the manuscript. RH assisted with coding, participated in design of the study and edited the manuscript. GR supervised this research, directed design of this study and the statistical analysis employed, and edited the manuscript. CMD conceived this piece of work, supervised the research, directed design of the study and edited the manuscript. The final manuscript was read and approved by all authors.

## Supplementary Material

Additional file 1 **Figure S1.** Surface *versus* buried prediction P-ROC curves for MI variants on the 40 test cases. A, B and C illustrate the performance of MI, MIp and MIc variants respectively when distinguishing surface from buried residues. The solid green line in all plots depicts the chance of randomly selecting surface residues, while the dashed green line indicates the probability of randomly selecting a surface residue when employing the reduced alphabet amino acid set. Click here for file

Additional file 2 **Figure S2.** Contact *versus* non-contact prediction MCC curves for MI variants on the 40 test cases. Performance evaluation of the predictive power of MI, MIp and MIc using the Matthews Correlation Coefficient (MCC) score [54]. A, B and C illustrate the performance of MI, MIp and MIc variants respectively when distinguishing contact from non-contact surface residues. The solid green line at 0 in all plots depicts the chance of randomly selecting a contact residue. An MCC score of + 1 indicates a perfect prediction, while a score of −1 represents total disagreement between prediction and observation. Click here for file
