# Supplementary figures and images for: Mutual information and variants for protein domain-domain contact prediction
Source: BMC Res Notes. 2012 Aug 31;5:472. doi: 10.1186/1756-0500-5-472 (PMC3532072; doi:10.1186/1756-0500-5-472)

**A.****MI**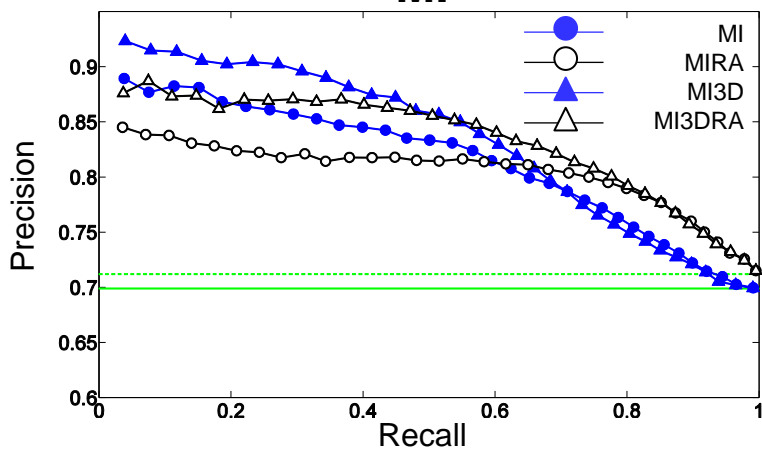**B.****Mlp**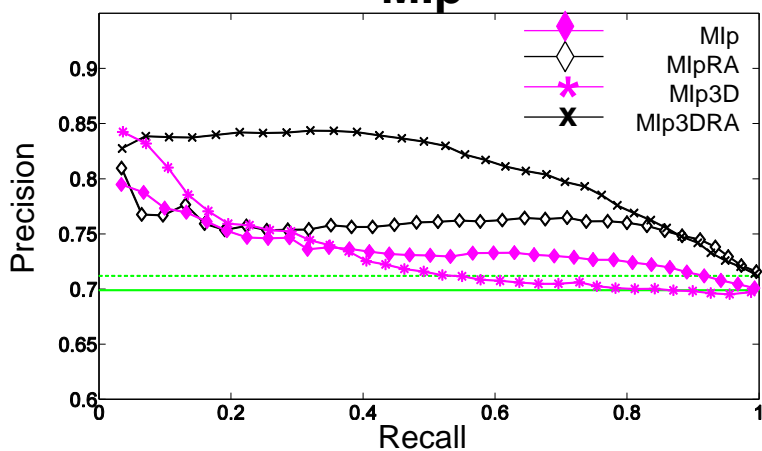**C.****Mlc**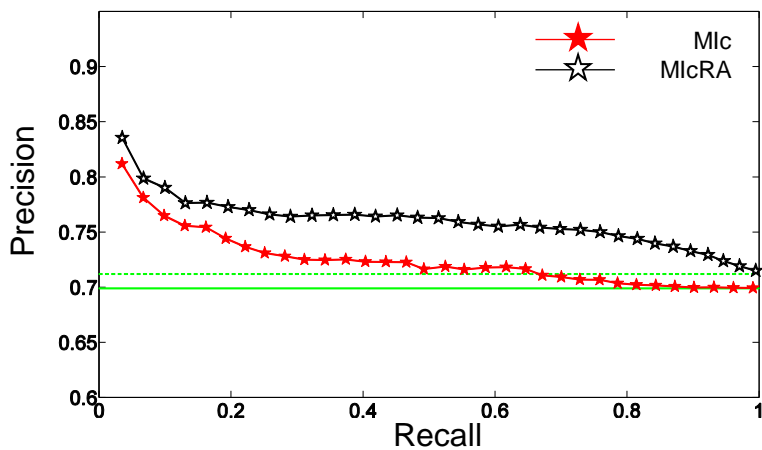

Supplement: Additional file 1 — Figure S1. Surface versus buried prediction P-ROC curves for MI variants on the 40 test cases. A, B and C illustrate the performance of MI, MIp and MIc variants respectively when distinguishing surface from buried residues. The solid green line in all plots depicts the chance of randomly selecting surface residues, while the dashed green line indicates the probability of randomly selecting a surface residue when employing the reduced alphabet amino acid set. [file 1756-0500-5-472-S1.pdf]

**A.****MI**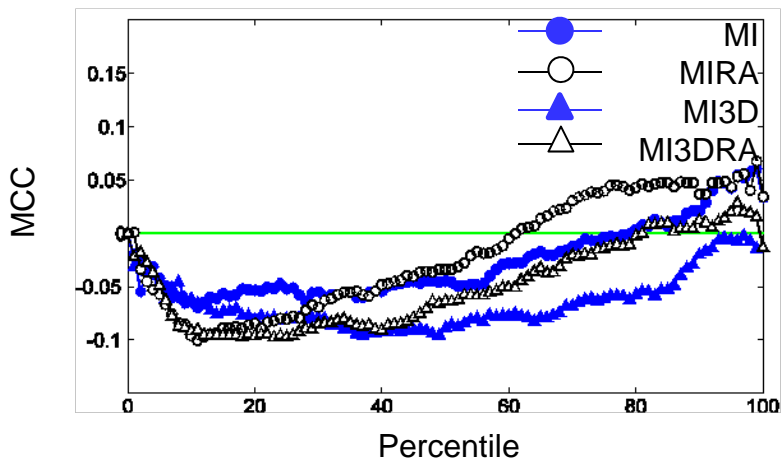**B.****Mlp**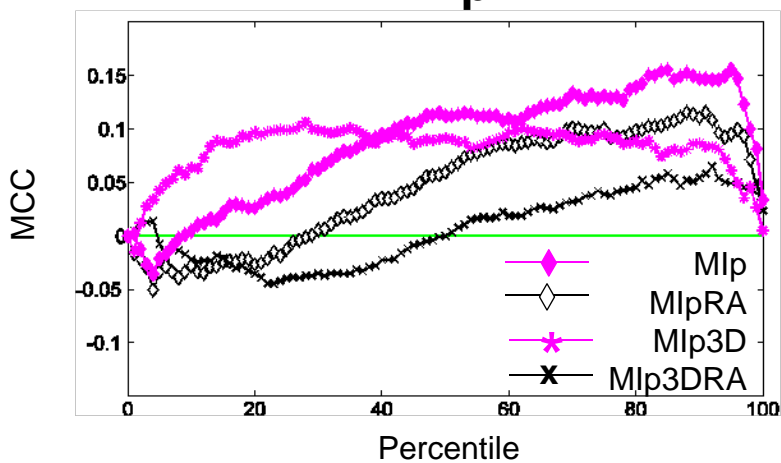**C.****Mlc**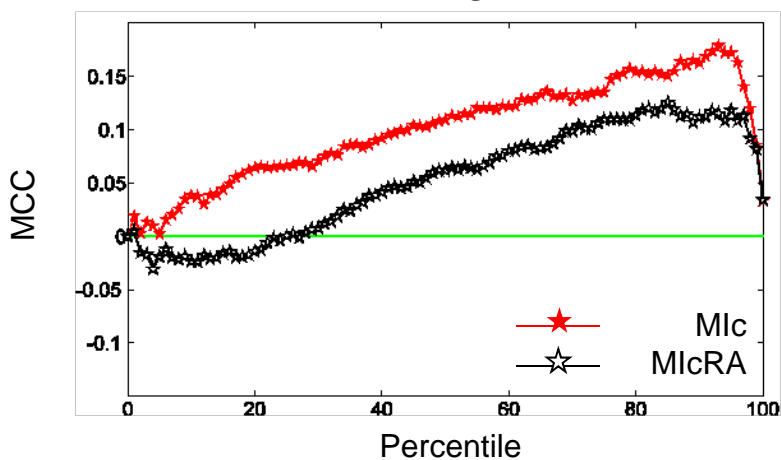

Supplement: Additional file 2 — Figure S2. Contact versus non-contact prediction MCC curves for MI variants on the 40 test cases. Performance evaluation of the predictive power of MI, MIp and MIc using the Matthews Correlation Coefficient (MCC) score [54]. A, B and C illustrate the performance of MI, MIp and MIc variants respectively when distinguishing contact from non-contact surface residues. The solid green line at 0 in all plots depicts the chance of randomly selecting a contact residue. An MCC score of + 1 indicates a perfect prediction, while a score of −1 represents total disagreement between prediction and observation. [file 1756-0500-5-472-S2.pdf]
